# Diagnostic challenge of perimenopause molar pregnancy in a 52-year-old lady: Case report

**DOI:** 10.1016/j.ijscr.2022.107648

**Published:** 2022-09-13

**Authors:** Willbroad Kyejo, Davis Rubagumya, Gregory Ntiyakuze, Nancy Matillya, Munawar Kaguta, Miriam Mgonja, Lynn Moshi

**Affiliations:** a Department of Family Medicine, The Aga Khan University, P.O. Box 38129, Dar Es Salaam, Tanzania; b Department of Family Medicine, Premier Care Clinic Masaki, PO Box 220, Dar Es Salaam, Tanzania; c Department of Obstetrics and Gynecology, The Aga Khan University, P.O. Box 38129, Dar Es Salaam, Tanzania

**Keywords:** Choriocarcinoma, Postmenopausal bleeding, Case report

## Abstract

**Introduction and importance:**

Gestational trophoblastic disease is an uncommon group of pregnancy-related disorders, with a course of trophoblastic proliferation, including hydatidiform mole (Agha et al., 2020), invasive and metastatic mole, choriocarcinoma, placental-site trophoblastic tumor, and epithelial trophoblastic tumor. Choriocarcinoma and trophoblastic tumor of the placenta are the most important tumors associated with pregnancy.

**Case findings:**

A 52-year-old woman Para 2 Living 3, 3 years post-menopausal presented with prolong per vaginal bleeding for five weeks accompanied by lower abdominal pain. Diagnosis of gestational trophoblastic disease (choriocarcinoma type) was made by using beta HCG, radiology, and histology report. Patient underwent total abdominal hysterectomy and bilateral salphingo-opherectomy, followed by 2 cycles of chemotherapy.

**Discussion:**

Trophoblast disease of pregnancy disease includes a unique tissue group with a wide range of endocrine and angiogenic functions derived from placental trophoblasts. They are associated with uncommon, interrelated conditions, which differ according to the following parameters: invasion, regression, metastasis, and recurrence rate. Beta HCG remains initial investigation to be taken in patients suspecting trophoblastic disease.

**Conclusion and recommendations:**

Gestational trophoblastic disease should be considered in the differential diagnosis of peri and postmenopausal vaginal bleeding. Long term follows up with beta HCG needs to be done to detect recurrence.

## Highlights

• Suspicion for Gestational Trophoblastic Disease in post-menopausal woman.
• General approach in unusual presentation of choriocarcinoma
• Multidisciplinary approach

## 1 Introduction

The trophoblast is a unique tissue, with special morphological and immunohistochemical characteristics related to its various metabolic, endocrine and angiogenetic functions [2]. Infiltration and metastasis are the main criteria for the diagnosis of a malignant tumor, but these criteria cannot be applied in the case of trophoblast, as they are normal features of this tissue, whose role is to infiltrate the endometrium, the underlying myometrium and parent vessels [2].

Trophoblast cells are an important parameter for the formation of placental tissue. Their role is to promote the penetration of the fetus inside the uterus to penetrate the uterine blood vessels, to induce angiogenesis and to produce human chorionic gonadotropin. Trophoblast is enrolled in three-layer types of cells: a) Cytotrophoblast or Langhans cells internal layer, b) intermediate trophoblastic cells mid layer and c) and an external layer of syncytiotrophoblastic cells [3].

Cytotrophoblastic cells are small, round mononuclear cells with distinct cell borders, minimal clear or eosinophilic cytoplasm and vesicular nuclei. Intermediate trophoblasts are larger than cytotrophoblasts, polygonal with abundant clear cytoplasm, distinct cell borders and single nuclei. Syncytiotrophoblasts are multinucleated giant cells with eosinophilic or basophilic cytoplasm and dense, large, pyknotic nuclei [4].

Syncytiotrophoblasts infiltrate the myometrium to ensure the successful implantation and proper nutrition of the fetus. This invasion process seems to be regulated mainly by the mechanism of apoptosis, which, in its turn, is controlled by the balance of two proteins belonging to the same protein family: bcl-2 protein, which demotes apoptosis and bax protein, which promotes it [5].

Gestational trophoblastic disease is an uncommon group of pregnancy-related disorders, with a course of trophoblastic proliferation, including hydatidiform mole (HM), invasive and metastatic mole, choriocarcinoma, placental-site trophoblastic tumor, and epithelial trophoblastic tumor [6].

Choriocarcinoma and trophoblastic tumor of the placenta are the most important tumors associated with pregnancy [7]. Choriocarcinoma is an extremely aggressive tumor that originates in the trophoblast of any gestational age [8].

Similar to pregnancy, the change from hCG production to hPL reflects and confirms the maturation of trophoblastic tissue [8]. The same theory applies to choriocarcinoma which is considered an immature tumor with very aggressive growth and early metastases [8].

Based on histological findings of the hysterectomy and bilateral oopherectomy, salpingectomy and HCG titer, in order to contribute to the scientific experience and further understand gestational choriocarcinoma, we reviewed the medical record of a 51-year-old woman with gestational choriocarcinoma, 30 years after her last normal deliver 3 years after her last menstrual period. This paper has been reported in line with the SCARE 2020 criteria [1]. This article has been registered with the Research Registry with identification number researchregistry8060 and can be found through the following hyperlink Browse the Registry - Research Registry.

## 2 Case presentation

A 52-year-old woman Para 2 Living 3 present in Family Medicine Clinic with prolong per vaginal bleeding for five weeks accompanied by lower abdominal pain. Attained menarche 3 years ago. Initially the bleeding was minimal, progressed to heavy flow soaking at least 4 maternity pads per day. She reported increase in lower abdominal girth reaching the umbilical level. Furthermore, she noticed to have lost weight and experienced heart burn, easy fatigue, shortness of breath as well as awareness of her heartbeats.

She denies any history of hot flushes or heat intolerance. She reported being seen in other facilities where the impression of symptomatic fibroid was made and she was given medication, however there was no relief.

She had regular menstrual cycle of 30 days duration lasting for 4 to 5 days. The menstrual flow was normal. Last delivery was 30 years ago, and she has history of using injectable contraceptives. However, she then used intrauterine contraceptive device (copper T), then changed to calendar method. She has been taking Primrose evening oil tablet daily for six months.

She otherwise had no significant family history of disease, no drug allergies and did not smoke or drink alcohol.

On admission she was pale and tachycardia, the abdomen was symmetrical distended, old healed SUMI scar, soft abdomen but firm uterine fundus palpable at umbilical level (20 cm), mild tenderness on the supra pubic, liver and spleen not enlarged, and kidneys were not ballotable.

Speculum reveal normal perineum, visible stained sanitary pad with blood, vaginal walls and cervix mucosa was health, a visible bleeding seen from the cervical external os. Other systemic examinations were essentially normal.

**Fig. 1:**
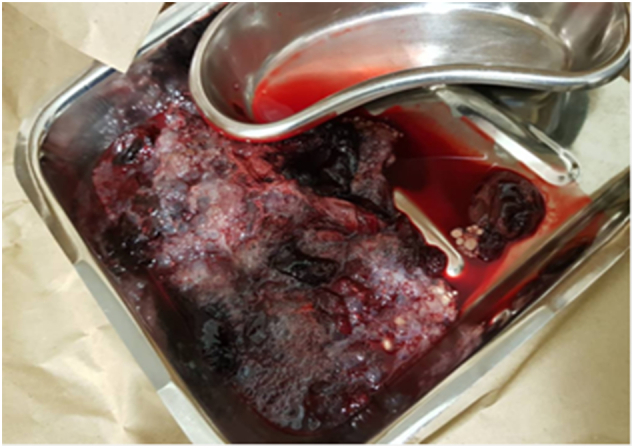
Uterine contents from the vaginal contained blood, multiple small vesicles mixed with minimal clots.

**Fig. 2:**
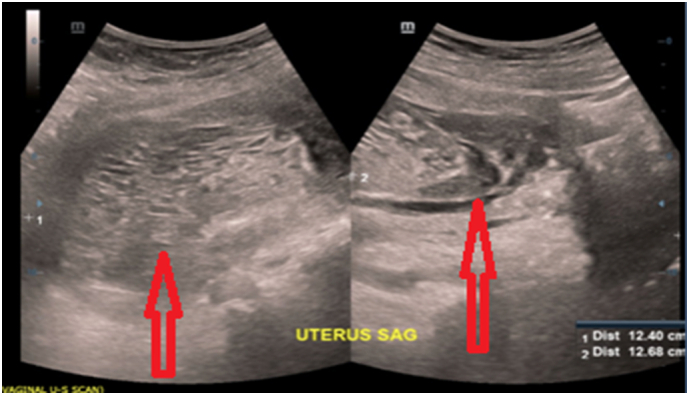
Below ultrasound scan reveal endometrium thickened of 5.2 cm bunch of grapes 13.3 × 6.8 cm, no vascularity appreciated.

Patient was admitted in the ward with diagnosis of bleeding fibroids while in the ward waiting for the ultrasound scan, she suddenly expelled uterine content with vesicles, which was later sent for histological examination (Fig. 1). Ultrasound scan reveal endometrium thickened of 5.2 cm, large hyperechoic heterogeneous lesion (bunch of grapes) with cystic changes which measure 13.3 × 6.8 cm, no vascularity appreciated (Fig. 2).

Initial labs results were as follows: White cell count of 11 × 10^9^/l, hemoglobin 11.1 g/dl, platelets 184 × 10^9^/l when compare with complete blood count post expulsion of uterine products white cell count of 10.2 × 10^9^/l, hemoglobin 9.4 g/dl and platelets were 114 × 10^9^/l.

Other laboratory parameters were CA 125 (serum) 44.78 U/ml (0–35.0), free T3 (serum) 8.11 pmol/l (3.10–6.8), free T4 (serum) 39.27 pmol/l (12.0–22.0), thyroid stimulating hormone (serum) 0.013 μIU/ml (0.270–4.20), FSH (serum) 0.100 mIU/ml <0.1 menopausal range, and beta HCG (serum) 680,058 mIU/ml <0.01 menopausal range.

From the laboratory parameters it highly suggestive of molar pregnancy with hyperthyroidism mimic elements from as it presented from levels of thyroid stimulating hormone and free T4 levels.

**Fig. 3:**
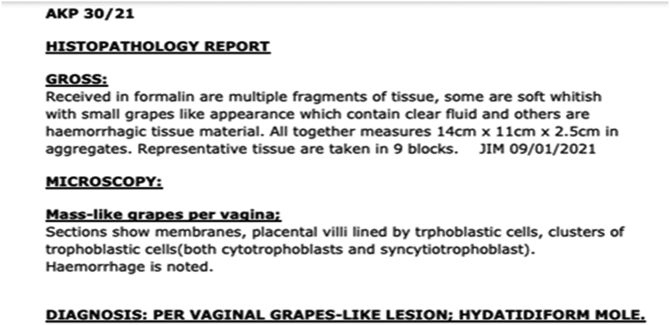
Below histology showing biopsy results from the uterine content products.

Histology results showed hydatiform mole (Fig. 3). Chest X ray and MRI of the abdomen were done to look for the sign of metastasis were done which were normal.

A final diagnosis of gestation trophoblastic tumor with a score of ≥7 (according to FIGO risk score and GTN staging) [9]. Tumor board decision was made to start EMA-CO (etoposide, methotrexate, actinomycin D, cyclophosphamide, vincristine) chemotherapy, but the patient refused due to fear of side effects of chemotherapy. However, later patient consented for total abdominal hysterectomy and bilateral salpingectomy and all organs were sent to lab. Post operative Oncologist teams were consulted and initiate 2 cycles of EMA-CO.

**Fig. 4:**
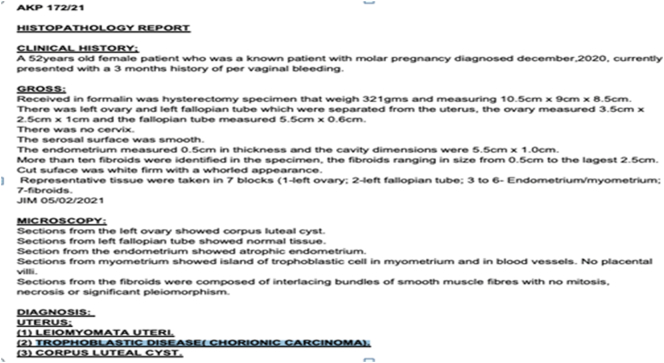
Below post total abdominal hysterectomy and bilateral salpingectomy organs biopsy.

Post total abdominal hysterectomy and bilateral salpingectomy biopsy showed leiomyomata uteri, trophoblastic disease (chorionic carcinoma) and corpus luteal cyst (Fig. 4).

**Fig. 5:**
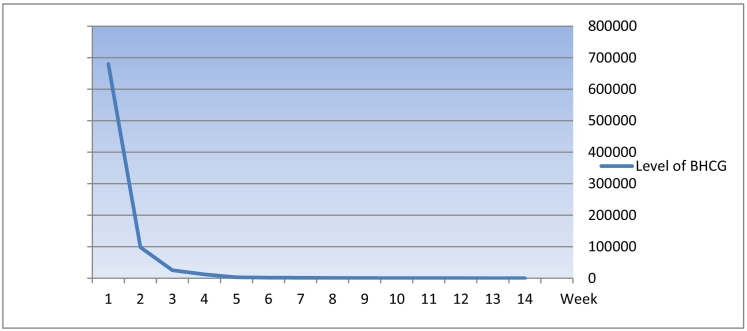
Below chart showing decrease of BHCG versus corresponding week of measure.

Beta human chorionic gonadotropin (BHCG) weekly follows up till 14th week is as shown in the graph (Fig. 5).

## 3 Discussion

A patient age at 52 years old obscured our attention on the possibility of neither normal pregnant nor molar pregnant. It is very rare to encounter such a case unless in a history of attempted intra vitro fertilization. This had led to delayed diagnosis and initiation of treatment in the first hospital she attended. Majority of perimenopausal or postmenopausal women do present in our setting with abnormal vaginal bleeding due to endometrial hyperplasia, endometrial cancer, and cervical cancer.

Trophoblast disease of pregnancy or gestational trophoblastic disease includes a unique tissue group with a wide range of endocrine and angiogenic functions derived from placental trophoblasts [10]. They are associated with uncommon, interrelated conditions, which differ according to the following parameters: invasion, regression, metastasis and recurrence rate [11].

Gestational trophoblastic neoplasia occurs most commonly in reproductive age after antecedent gestational events, like recent pregnancy usually within a year of a preceding pregnancy or more decades in the past, molar or non-pregnancy in 50 % of the cases, abortions, ectopic pregnancies also recently or after a long time in the past, while only a few cases have been reported in the international literature [7], [17]. In complete molar pregnancies the classical occurrence of large edematous clusters of vesicles with hemorrhage and necrosis is typical. The majority karyotype of complete molar pregnancies are 46XX in 95 % of cases arise from fertilization of an empty pronucleus by haploid sperm that undergoes duplication, Diandric diploidy, while the minority are 46 XY in 5 % of cases which arise from fertilization of an empty egg by two sperm and led to Diandric dispermy. Partial mole is associated to less abundant hydropic villi large edematous with stromal fibrosis [12].

Gestational trophoblastic disease is the appropriate collective name but interrelated pathological conditions derived from placental trophoblasts for partial, complete molar pregnancy (both characterized by the presence of hydropic villi which usually contain fetal vessels and fetal red blood cells), miscellaneous trophoblastic tumors (exaggerated placental site, placental site nodule or plaque),unclassified trophoblastic lesions, choriocarcinoma and placental site trophoblastic tumor [11], [17].

Gestational choriocarcinoma can occur by any gestational event as follows: after hydatidiform mole in 50 % of cases, 25 % after spontaneous abortion, 22.5 % after normal pregnancy and 2.5 % after ectopic pregnancy (1 in 5333 tubal pregnancies) [13]. Not always to the predisponding pregnancy within the uterine cavity is referred as one of the most related factors within a year of the referred pregnancy [13]. Especially in the premenopausal women gestational trophoblastic pathology is subsequent hydatid mole in 60 % of cases, abortions in 30 % and in 10 % normal or ectopic pregnancy. While in Europe and North America the occurrence is 30,000–40,000 intrauterine and 1/40 molar pregnancies, the rates increase to 1/500–3000 in south East Asia [13]. In Sub Saharan African countries has limited data in gestational choriocarcinoma.

Tsukamoto et al. reported 20 women of GTD (≥50 years), none of whom were diagnosed with complete hydatiform molar. In China, Feng et al. reviewed 38 cases of GTD in women aged 50 years or more, of whom 19 had invasive moles, five were HM patients, 12 were choriocarcinomas patients, and two had placenta-site trophoblastic disease [14].

There are about 14 cases [15] in the global literature concerning choriocarcinoma in the postmenopausal period. Based on these cases, the most common symptoms of choriocarcinoma in postmenopausal women are vaginal bleeding (approximately 80 %), uterine enlargement (100 %), abdominal pain (42 %), and nausea and vomiting (25 %), as well as markedly elevated serum β-HCG level. This is similar as it presented in our case.

The choriocarcinomas, which are highly vascular neoplasms have as main clinical presentation abnormal uterine bleeding and metastasize readily in approximately 30 % of cases at the time of the final diagnosis and most commonly in lung 60–75 %, vagina 40–50 %, brain 15–20 %, liver 15–20 %, spleen 10 %, intestines 10 % (5 % bowel metastasis), and central nervous system involvement in 10 % of cases, very rare manifestation, 4 % in cardiac intracavity mass, cardiac metastasis [14], [16]. In our case, we did MRI of the abdomen and chest X ray which turn to be normal, and patient had no sign of metastasize.

Initial diagnosis is based on multimodality approach like considering clinical features; serial beta HCG titers and pelvic imaging-like ultrasonography and MRI. The most possible reason for delayed diagnosis of GTD in perimenopausal age group could be the absence of suspicion of pregnancy in them. Evidence suggests that incidence of choriocarcinoma had decreased due to strict follow up of patients with molar pregnancy [16]. This is similar to our case were the initial beta HCG were 680,058 mIU/ml which followed by spontaneous expulsion of vesicle like product from the vagina thus lead to diagnosis of gestational trophoblastic disease to be high in the list.

Ultrasound is first line of imaging. The appearance of lesion is classically described as snowstorm/granular appearance due to multiple echogenic foci [16]. This is similar to our case whereby initial ultrasound showed thickened endometrial wall and bunch of grades in the uterine cavity which is highly suggestive of gestational trophoblastic disease.

Pelvic MRI is used to assess depth of myometrial invasion and extra uterine spread in equivocal and complicated cases. Chest radiography, PET CT or brain MRI has been recommended as investigative tools for overall staging of the disease. Angiography has a role in management of disease complications with metastasis [13], [16].

In our case, the indication of confirmation gestational choriocarcinoma was based on high β-hCG serum levels, radiology findings and histological evident findings comprising of leiomyomata uteri, chorionic carcinoma, and corpus luteal cyst. Due to high-risk score and early stage according to FIGO staging we recommended EMA-CO chemotherapy regime, but initially patient refused due to fear of side effects of chemotherapy however later patient was sent for total abdominal hysterectomy and bilateral salphio-oopherectomy and later receive 2 cycle of EMA-CO this decision was based on international oncology treatment protocols. Post chemotherapy level of beta HCG decrease dramatically to undetectable level in week 14 after surgery and 2 cycle of chemotherapy.

This reported case is of clinical importance due to the patient's long 30-year period after her last gestational event age of patient 3 years postmenopausal long time after last gestational event. Generally, the prognostic value in reproductive time of the long duration between last gestation event and tumor diagnosis is negative associated to poor prognosis, however in the postmenopausal time unknown, possible poor.

## 4 Conclusion and recommendation

GTD should be considered in the differential diagnosis of peri and postmenopausal vaginal bleeding. In menopausal women, definitive treatment should be hysterectomy owing to high risk of malignancy. A high level of suspicion should be included in differential diagnosis of peri menopausal bleeding to prevent delay in diagnosis and treatment. Long term follows up with beta HCG needs to be done to detect recurrence.

## Abbreviations

FIGO: International Federation of Gynecology and Obstetric
GTD: Gestation trophoblastic disease
BHCG: Beta human chorionic gonadotropin hormone
MRI: Magnetic resonance intensity
SUMI: Sub umbilical midline incision

## Provenance and peer review

Not commissioned, externally peer-reviewed.

## Consent

Written informed consent was obtained from the patient for publication of this case report and accompanying images. A copy of the written consent is available for review by the Editor-in-Chief of this journal on request.

## Ethical approval

Case study is exempt from ethical approval in my institution.

## Funding

This research did not receive any specific grant from funding agencies in the public, commercial, or not-for-profit sectors.

## Guarantor

Dr. Lynn Moshi, Obstetric and Gynecologist, Aga Khan Hospital.

## Research registration number

1. Name of the registry: RESEARCH REGISTRY
2. Unique identifying number or registration ID: researchregistry8060
3. Hyperlink to your specific registration (must be publicly accessible and will be checked): https://www.researchregistry.com/register-now#user-researchregistry/registerresearchdetails/62487eb5de369c001ebba679/.

## CRediT authorship contribution statement

W.K.: Study conception, production of initial manuscript, collection of data
D.R.: Production of initial manuscript, revision of the manuscript, proofreading
G.N.: Revision of the manuscript, proofreading
M.M.: Production of initial manuscript, collection of data
N.M.: Revision of the manuscript, proofreading
M.K.: Study conception, revision of the manuscript, proofreading
L.M.: Study conception, revision of the manuscript, proofreading.

## Declaration of competing interest

The author declares no potential conflicts of interest with respect to the research, authorship, and/or publication of this article.
